# Effects of periodontal pathogen-induced intestinal dysbiosis on transplant immunity in an allogenic skin graft model

**DOI:** 10.1038/s41598-023-27861-4

**Published:** 2023-01-11

**Authors:** Takanori Mei, Hiroshi Noguchi, Ryutaro Kuraji, Shinsuke Kubo, Yu Sato, Keizo Kaku, Yasuhiro Okabe, Hideya Onishi, Masafumi Nakamura

**Affiliations:** 1 Department of Surgery and Oncology, Graduate School of Medical Sciences, Kyushu University, 3-1-1 Maidashi, Fukuoka, 812-8582 Japan; 2 Department of Periodontology, The Nippon Dental University School of Life Dentistry at Tokyo, Tokyo, Japan; 3 Department of Cancer and Research, Graduate School of Medical Sciences, Kyushu University, Fukuoka, Japan

**Keywords:** Immunology, Medical research, Dental diseases, Immunological disorders, Oral diseases

## Abstract

Periodontal disease can induce dysbiosis, a compositional and functional alteration in the microbiota. Dysbiosis induced by periodontal disease is known to cause systemic inflammation and may affect transplant immunity. Here, we examined the effects of periodontal disease-related intestinal dysbiosis on transplant immunity using a mouse model of allogenic skin graft in which the mice were orally administered the periodontal pathogen *Porphyromonas gingivalis* (*Pg*). For 6 weeks, the *Pg* group orally received *Pg* while the control group orally received phosphate-buffered saline solution. After that, both groups received allogenic skin grafts. 16 s rRNA analysis of feces revealed that oral administration of *Pg* significantly increased three short chain fatty acids (SCFAs) producing genera. SCFA (acetate and propionate) levels were significantly higher in the *Pg* group (*p* = 0.040 and *p* = 0.005). The ratio of regulatory T cells, which are positively correlated with SCFAs, to total CD4+ T cells in the peripheral blood and spleen was significantly greater (*p* = 0.002 and *p* < 0.001) in the *Pg* group by flowcytometry. Finally, oral administration of *Pg* significantly prolonged skin graft survival (*p* < 0.001) and reduced pathological inflammation in transplanted skin grafts. In conclusion, periodontal pathogen-induced intestinal dysbiosis may affect transplant immunity through increased levels of SCFAs and regulatory T cells. (198 words).

## Introduction

Transplantation is a common treatment for end-stage organ failure. However, most transplant recipients require lifelong immunosuppressive drugs to prevent acute immune-mediated rejection of donor organs, making them susceptible to infection, malignancy, and drug toxicity^1–3^. Even in patients who receive immunosuppressive drugs, acute graft rejection can occur, preventing successful long-term engraftment^4^. Transplant recipients show heterogeneity in the occurrence and timing of acute rejection^5–7^. Understanding the factors that contribute to this heterogeneity may improve graft survival.

Several clinical studies have reported an association between transplant immunity and periodontal disease. Nunes-dos-Santos et al.^8^ showed that there is a positive association between periodontal status and worsening of graft function among kidney transplant recipients. Conversely, Min et al.^9^ found that the presence of severe periodontitis was independently associated with a lower incidence of acute T cell-mediated rejection in kidney transplant recipients, suggesting a possible effect of periodontitis on immune function. Although the mechanism by which periodontal disease affects transplantation immunity remains unclear, periodontitis may be a cause of heterogeneity in transplant immunity.

Periodontitis is a common infectious, chronic, inflammatory disease that is caused by changes in the oral microbial biofilm^10–12^. Periodontitis, which is characterized by inflammatory bone resorption of the teeth-supporting structures, is the most prevalent form of bone pathology, leading to periodontal tissue destruction and tooth loss^13^. Moreover, periodontitis is associated with the development or exacerbation of various systemic diseases, including obesity, diabetes, nonalcoholic liver disease, and cancers^14–17^. One of the proposed mechanisms by which periodontal disease induces systemic inflammation is alteration of gut microbial composition by swallowed periodontal bacteria^18^. Periodontal disease can induce ‘dysbiosis’, which is defined as a compositional and functional alteration in the microbiota that is driven by a set of environmental and host-related factors^19^.

The gut microbiota is a complex microbial ecosystem, and maintaining the mutualistic relationship between the gut microbiota and host is critical for human health^20^. The gut microbiota is also likely to play a significant role in the immune responses that determine the fate of transplanted allografts, leading to tolerance or rejection^21^. Several potential mechanisms have been suggested. One of these mechanisms is thought to be mediated by short-chain fatty acids (SCFAs), which are fecal microbiome metabolites. SCFAs such as butyrate and propionate have been reported to induce regulatory T cells (Tregs)^22^. Because Tregs are known to inhibit graft rejection^23^, SCFAs may affect transplant immunity.

Humans swallow about 1.5 L of saliva daily, along with millions of oral microbes^24^. In general, it had been believed that more than 99% of oral microbes die as they pass through the acidic environment of the stomach and later the small intestine, which act as a barrier against bacteria from the mouth and gut. But Schmidt et al.^25^ reported that a vast majority of oral species can transfer to and subsequently colonize the intestine, and that oral-fecal transmission is an important process that shapes the gastrointestinal microbiome. Arimatsu et al.^18^ also showed that oral administration of *Porphyromonas gingivalis* (*Pg*), a typical periodontal pathogen, induces systemic inflammation and metabolic changes via dysbiosis in mice.

In this study, we examined the effects of periodontal pathogen-induced intestinal dysbiosis on transplant immunity using a mouse model of allogenic skin graft in which the mice were orally administered *Pg*.

The purposes of this study were to examine the effects of periodontal pathogen-induced intestinal dysbiosis on transplant immunity using a mouse model of allogeneic skin transplant in which the mice were orally administered *Pg* and to elucidate the molecular mechanisms by which fecal microbiome metabolites and Tregs mediate the relationship between periodontal disease and transplant immunity.

## Results

### Oral administration of *Pg* increased the relative abundance of short chain fatty acid-producing genera

**Figure 1:**
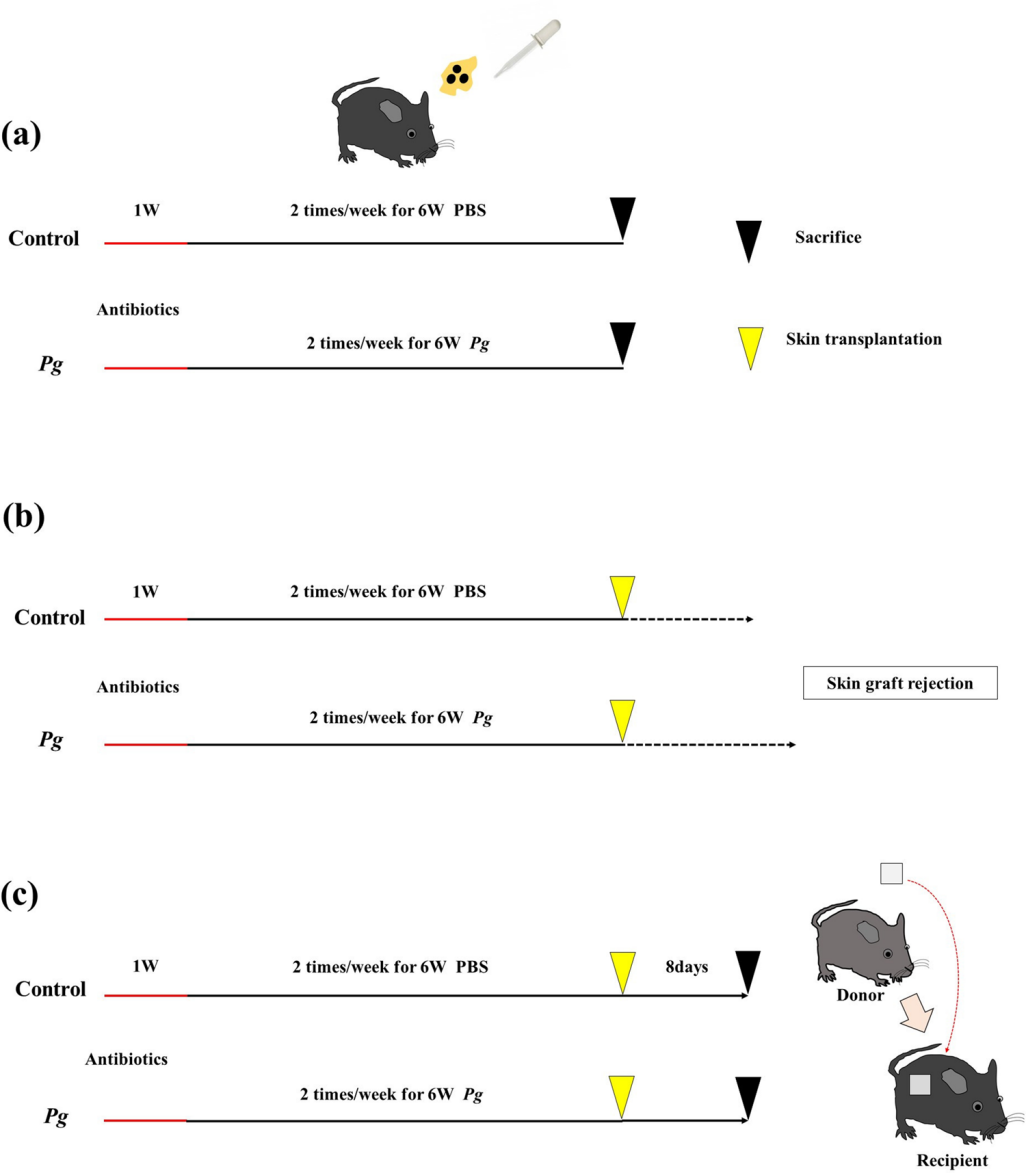
Study design. All mice used in this experiment were given antibiotics in their drinking water for one week. After that, mice in the *Porphyromonas gingivalis* group received *Pg* orally twice per week for 6 weeks, while mice in the control group received PBS orally twice per week for 6 weeks. (**a**) Both groups were sacrificed before transplantation. (**b**) Both groups received allogenic skin grafts and were monitored until skin graft rejection. (**c**) Both groups received allogenic skin grafts. On day 8 after skin grafting, the mice in both groups were sacrificed.

To investigate the effects of periodontal disease-related intestinal dysbiosis on transplant immunity, we divided the mice into two groups: the *Pg* group and the control group. Mice in the *Pg* group received *Pg* orally twice per week for 6 weeks, whereas mice in the control group received phosphate-buffered saline solution (PBS) orally twice per week for 6 weeks. Then, both groups received allogenic skin grafts. Figure 1 depicts the study design. To examine microbiome changes between the groups, we collected fecal samples from the cecum before transplantation and conducted 16 s rRNA sequencing analysis to determine the taxonomy of the microbes in both groups. β-diversity analysis, which is generally used to identify variation in the identities of species among samples^26^, was also performed to evaluate changes in diversity in both groups.

**Figure 2:**
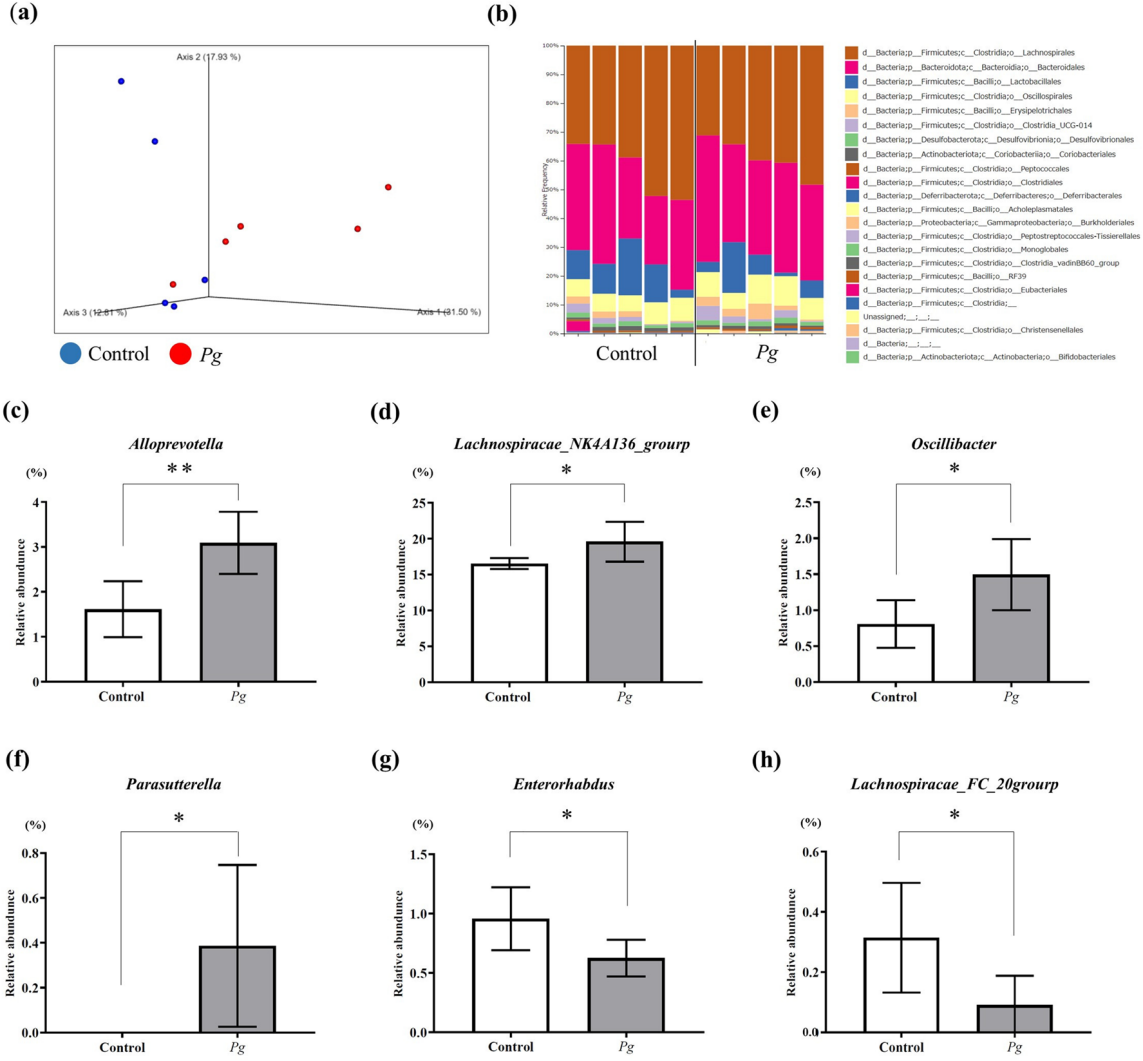
Analysis of the microbiota in both groups. (**a**) Unweighted UniFrac distances between the groups (red: *Pg*; green: control). (**b**) Taxonomic bar plots at the order level for both groups. (**c**–**h**) There was a significant change in the relative abundance of six genera between groups (four genera increased, and two genera decreased). (**c**) *Alloprevotella*, (**d**) *Lachnospiracae_NK4A136_group*, (**e**) *Oscillibacter,* (**f**) *Parasutterella*, (**g**) *Enterorhabdus,* (**h**) *Lachnospiracae_FCS020_group.* Each group: n = 5. *p < 0.05, ** *p* < 0.005.

The observed features values, indicators of α-diversity, showed a tendency to decrease in the Pg group compared with the control group, but did not change significantly (Supplementary Fig. S1). As shown in Fig. 2a, unweighted UniFrac values were plotted as an indicator of β-diversity by means of principal coordinates analysis. Similar to the results of α-diversity, the unweighted UniFrac values for the Pg group showed a trend toward a separate population from the control group, but there was no significant difference between the groups (*p* = 0.181).

Figure 2b shows the composition of microbiota at the order level. At the genus level, 87 genera were detected in both groups, and there was a significant difference in the relative abundance of six genera between the groups (Fig. 2c–h). The relative abundances of *Alloprevotella* (*p* = 0.007), *Lachnospiraceae_NK4A136_group* (*p* = 0.042), *Oscillibacter* (*p* = 0.033), and *Parasutterella* (*p* = 0.043) were significantly greater in the *Pg* group than in the control group. In contrast, the abundances of *Lachnospiraceae_FCS020_group* (*p* = 0.046) and *Enterorhabdus* (*p* = 0.042) were significantly lower in the *Pg* group than in the control group. These results indicate that the abundance of SCFA-producing genera^27,28^ (*Alloprevotella*, *Lachnospiraceae_NK4A136_group*, and *Oscillibacter*) were significantly increased in the *Pg* group compared with the control group.

### Oral administration of *Pg* significantly increased the levels of SCFAs in the feces

**Figure 3:**
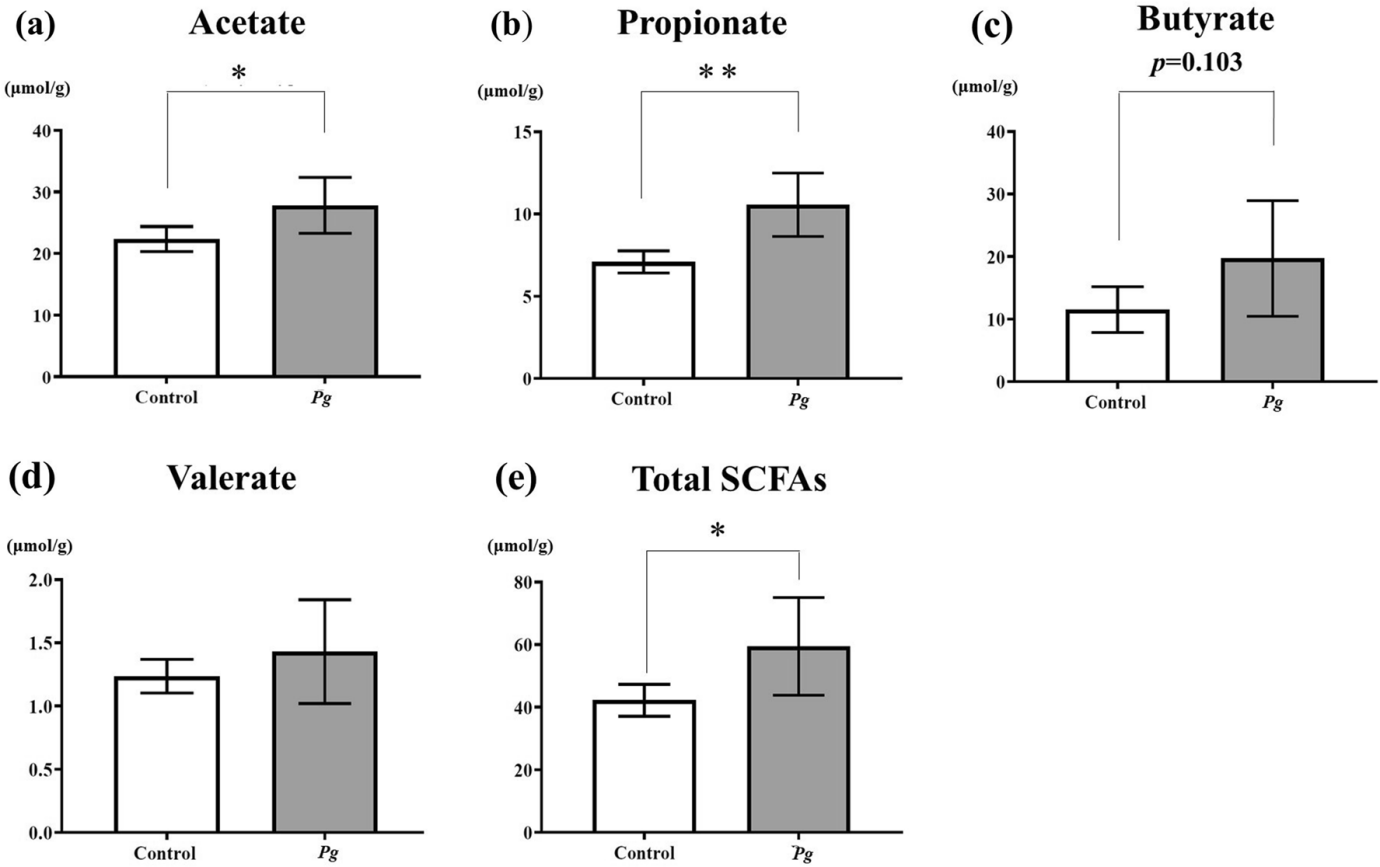
SCFAs in both groups. Short-chain fatty acid (SCFA) levels in the feces of mice from the *Pg* group and the control group, as determined by gas chromatography (each group: n = 5). (**a**) Acetate, (**b**) propionate, (**c**) butyrate, (**d**) valerate, (**e**) total SCFAs (total concentrations of acetate, propionate, butyrate, valerate, and caproate). *p < 0.05, **p < 0.005.

Because the abundance of SCFA-producing genera was increased in the *Pg* group, we measured levels of SCFAs, which are fecal microbiome metabolites, by gas chromatography in the *Pg* and control groups (Fig. 3).

Acetate levels were significantly higher in the *Pg* group (27.8 ± 4.5 µmol/g, *p* = 0.04) than in the control group (22.4 ± 2.0 µmol/g). Propionate levels were also significantly higher in the *Pg* group (10.6 ± 1.9 µmol/g, *p* = 0.005) than in the control group (7.1 ± 0.7 µmol/g). The butyrate level was about 1.7-fold greater in the *Pg* group (19.7 ± 9.3 µmol/g) than in the control group (11.5 ± 3.7 µmol/g), but this difference was not significant (*p* = 0.103). Valerate levels were slightly higher in the *Pg* group (1.4 ± 0.4 µmol/g, *p* = 0.344) than in the control group (1.2 ± 0.1 µmol/g). Caproate was not detected in either group. Taken together, total SCFA levels (which included the total concentration of acetate, propionate, butyrate, valerate, and caproate) were significantly higher in the *Pg* group (59.5 ± 15.6 µmol/g, *p* = 0.047) than in the control group (42.2 ± 5.1 µmol/g).

### Oral administration of *Pg* increased the proportion of Tregs out of total CD4+ T cells in the peripheral blood and spleen

**Figure 4:**
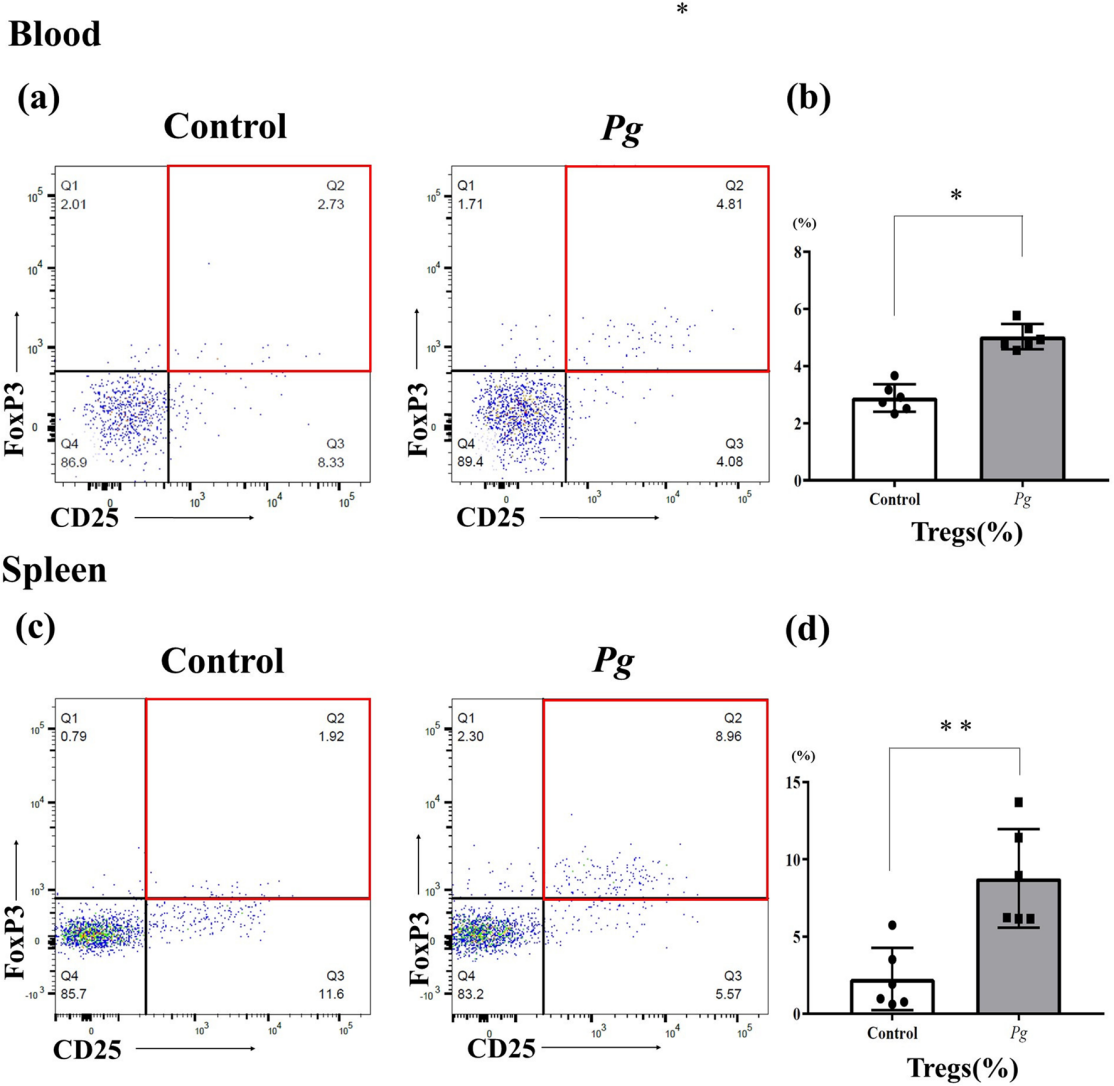
Flow cytometry analysis of cells from both groups. The proportion of Tregs out of CD4+ T cells in the peripheral blood (**a**) and spleen (**c**) in the *Pg* group and the control group before transplantation, as determined by flow cytometry (each group: n = 6). Statistical analysis was performed to identify differences between the groups. (**b**) Peripheral blood, (**d**) spleen. *p < 0.05, **p < 0.005.

To determine whether the increased level of SCFAs in the *Pg* group affected the proportion of Tregs, we determined the proportion of Tregs (CD25 + /forkhead box P3 (FoxP3) +) out of CD4 + T cells in the peripheral blood and spleen in the *Pg* group and the control group by flow cytometry (Fig. 4). In the peripheral blood, the proportion of Tregs was significantly greater in the *Pg* group (5.03 ± 0.44%, *p* = 0.002) than in the control group (2.88 ± 0.48%). Similarly, in the spleen the Treg proportion was significantly greater in the *Pg* group (8.77 ± 3.20%, *p* < 0.001) than in the control group (2.26 ± 2.01%).

### Oral administration of *Pg* prolonged skin graft survival

**Figure 5:**
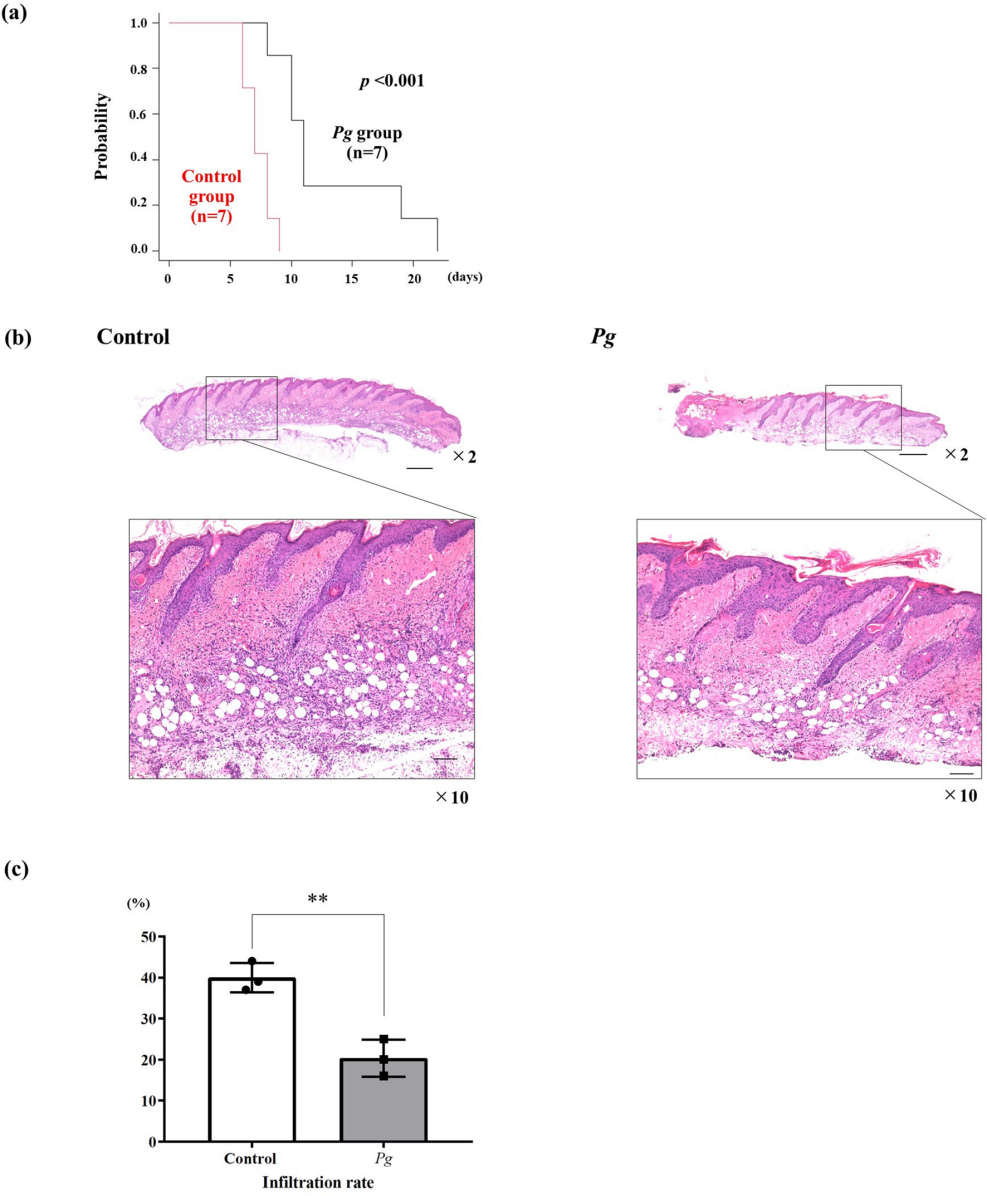
Skin graft survival and pathological changes in skin grafts in both groups. (**a**) Skin graft survival between the groups (each group: n = 7). On day 8 after skin grafting, the degree of inflammation of the skin graft was evaluated pathologically (hematoxylin/eosin staining). (**b**) × 2 magnification. The bar scale is 500 µm, × 10 magnification The bar scale is 100 µm. (**c**) Percentage of inflammatory cell infiltration in both groups (each group: n = 3). *p < 0.05, **p < 0.005.

To determine whether the increased proportion of Tregs out of total CD4 + T cells in the *Pg* group affected skin graft survival, allogenic mouse skin transplantation was performed in both groups (Figs. 1b, 5a). On one hand, in the control group, median skin graft survival was 7 days (range: 6–9 days). On the other hand, median skin graft survival was 11 days in the *Pg* group (range: 8–22 days). Oral administration of *Pg* significantly prolonged skin graft survival (*p* < 0.001).

### Oral administration of *Pg* reduced inflammation in transplanted skin

The degree of inflammation in the skin grafts was evaluated histopathologically (hematoxylin/eosin staining: H&E) on day 8 after skin grafting. The skin grafts in the *Pg* group exhibited less inflammation than those in the control group (Figs. 1c, 5b). As shown in the Fig. 5c, the *Pg* group exhibited a significantly lower degree of inflammatory cell infiltration compared with the control group (*p* < 0.001).

## Discussion

Oral administration of *Pg* can induce dysbiosis, which is associated with impaired gut barrier function, resulting in endotoxemia^18,29^. Periodontal bacteria-induced dysbiosis also may affect systemic immunity. For instance, in the collagen-induced mouse model of arthritis, oral administration of *Pg* induced gut dysbiosis and shifted the gut immune system toward a Th17-dominated response, resulting in aggravation of the collagen-induced arthritis^30^. However, to our knowledge, there are no reports of the relationship between periodontal bacteria-induced dysbiosis and transplant immunity. In the present study, we found that oral administration of *Pg* altered the gut microbiota, resulting in an increase in SCFA-producing genera and higher levels of SCFAs in the feces compared with the control mice. Also, the proportion of Tregs out of total CD4+ T cells was higher in the blood and spleen of mice in the *Pg* group. Furthermore, oral administration of *Pg* significantly prolonged skin graft survival. Thus, periodontal pathogen-induced intestinal dysbiosis may affect transplant immunity.

In a review of dysbiosis and the immune system, Levy et al.^19^ reported a common definition of dysbiosis, described as a compositional and functional alteration in the microbiota that is driven by a set of environmental and host-related factors. In the current study, oral administration of *Pg* induced significant changes of six genera (a compositional alteration). Moreover, three increased genera were SCFA-producing genera, consistent with the increased levels of SCFAs. Because SCFAs have known positive relationships with Tregs^22,31,32^, it is likely that a functional alteration also occurred in the *Pg* group. These changes were induced by oral administration of *Pg*, so we propose that intestinal dysbiosis occurred in the *Pg* group. Interestingly, Schmidt et al.^25^ reported that oral bacteria could transmit to and also colonize the gut. Colonization in the gut may change the composition of microbiota. Arimatsu et al.^18^ also reported that oral administration of *Pg* induced dysbiosis, but they did not detect *Pg* in feces of the small intestine. Therefore, Pg colonization in the gut and associated changes, rather than Pg per se, may induce dysbiosis. In the current study, there was no significant difference in the β diversity of the gut microbiome between the groups (Fig. 2). Chatelier et al.^33^ reported that diversity correlates with metabolic markers, and individuals with lower diversity are characterized by more marked overall adiposity, insulin resistance, dyslipidemia, and a more pronounced inflammatory phenotype. However, diversity is not always an accurate indicator of host status. Zhao et al.^34^ reported that greater overall gut microbiome diversity may not be as important for maintaining health as the balance among specific bacterial species, because consumption of a high-fiber diet by patients with type 2 diabetes was shown to improve HbA1c and fasting blood glucose, despite causing a significant reduction in overall diversity. Matson et al.^35^ showed that any one specific bacterial species may not be sufficient to determine immunity, but that the balance among beneficial and nonbeneficial species may be the most important determinant of clinical outcomes. The definition of dysbiosis by Levy et al.^19^ does not include diversity changes (for instance, loss of diversity), which is only one aspect of dysbiosis. Wu et al.^21^ also found that the relative abundance of bacterial species known to produce SCFAs, including *Bifidobacterium, Clostridium,* and *Bacteroidetes* spp., were significantly increased in mice with high levels of SCFAs. Thus, the increase in the relative abundance of the three SCFA-producing genera observed in our study may be related to the increased level of SCFAs observed in the feces.

Consistent with the gut microbial data, the level of SCFAs in the feces was higher in the *Pg* group than in the control group (Fig. 3). SCFAs in the intestine have been reported to induce colonic Tregs by inhibiting histone H3 deacetylation, which results in derepression of the regulatory region of the Foxp3 gene in vitro and in vivo^8^. SCFAs can also induce colonic and peripheral Treg expansion through GPR43 expressed on colonic T cells^31,32^. Moreover, SCFAs have the potential to regulate tissue inflammation through their effects on multiple cell types such as Tregs, dendritic cells, and macrophages^36^. Gao et al.^37^ reported that, in a rat model of experimental periodontitis induced by silk thread ligation in which *Pg* was applied around the molars, the proportion of Tregs in the peripheral blood was increased. These findings strongly support the relationship between the overproduction of SCFAs and the increase in the proportion of Tregs seen in the *Pg* group in our study (Fig. 4).

Moreover, we found that the median skin graft survival was significantly longer in the *Pg* group (Fig. 5). Wu et al.^21^ reported that, in a mouse model of kidney transplant, increased SCFA production caused by consumption of a high-fiber diet led to Treg development and prolonged allograft survival. Moreover, they reported that the administration of acetate, which is one of the SCFAs, significantly attenuated rejection of the graft and prolonged graft survival. Furthermore, Masetti et al.^38^ reported that in a allogeneic hematopoietic stem cell transplantation model, the administration of two main SCFAs, butyrate and propionate, in wild-type mice reduced acute graft versus host disease. Miao et al.^39^ also reported that oral administration of the probiotic bacterium *Lactobacillus rhamonosus* HNOO1 increased the level of the SCFA propionate in the feces and enriched Tregs in the spleen and in cardiac graft tissue, resulting in prolonged cardiac transplant survival. Importantly, in our study, the degree of inflammation in the skin graft on day 8 after skin grafting was significantly milder in the *Pg* group than in the control group.

The saliva swallowed by patients with periodontitis has been reported to contain up to 10^9^ bacteria/mL, in 1.0–1.5 L/day^40–42^, resulting in a total of greater than 10^12^ bacteria/day. The number of bacteria administered in the current study was determined by considering mouse body weight and the number of bacteria in the saliva of patients with periodontitis^18,29,43^. However, whether the amount of SCFAs in feces provides a dose-dependent effect on survival of skin grafts was not determined in the current study. Further studies are needed in the future.

Interestingly, the milieu that is good for the graft is not necessarily good for the host. Clinically, Noguchi et al.^44^ reported that sarcopenia, which is a syndrome characterized by progressive and generalized loss of skeletal muscle mass and strength^45^, is an independent predictive factor for better graft survival in pancreas transplant recipients. Blach et al.^46^ reported that periodontitis did not increase the risk of graft loss, but did increase the risk of patient death after kidney transplantation. They also showed that the hazard ratio for death was 7.17 for patients with periodontitis. Thus, the conflicting needs and complex statuses of the transplant graft and the host sometimes cause a ‘paradox of transplantation’.

To our knowledge, this is the first study to report the effect of periodontal pathogen-induced intestinal dysbiosis on transplant immunity via SCFAs and Tregs. However, our study had some limitations. Although the induction of intestinal dysbiosis through hematogenous spread of inflammatory cytokines and periodontal pathogenic bacteria and enteric translocation of oral bacteria have been proposed as pathways by which periodontal disease induces systemic inflammation^18^, we demonstrated only one mechanism by which oral administration of *Pg* can affect transplantation immunity through inducing changes in the gut microbiota and its metabolites. In addition, in the mouse model used in this experiment, intestinal dysbiosis was induced by oral administration of *Pg*, a typical periodontopathic bacterium, rather than by periodontitis more generally. Whether these observed effects on transplant immunity is *Pg*-specific or not has not been determined in the present study. Periodontal disease is essentially an inflammatory disease involving polymicrobial infection, and the beneficial effect of *Pg* infection on transplantation is not necessarily relevant to all microbes involved in periodontitis; indeed, previous studies have shown that periodontal disease-related dysbiosis can have a negative effect on transplant immunity and graft survival. How periodontal disease and polymicrobial infections affect transplantation immunity is a major question for the future.

Nevertheless, given the importance of managing transplant immunity to patient survival, and the high prevalence of periodontal disease worldwide, the results from this study could suggest novel clinical and public health strategies for transplant recipients with periodontitis. Conventional periodontal therapy and microbiome-targeted therapy using probiotics and antibiotics may be potential approaches that help improve transplant outcomes by improving the gut environment and transplant immunity. In addition, the present study investigated a skin transplant model, not a solid organ transplant model; hence, whether periodontitis has a similar impact on solid organ transplantation, such as kidney or liver transplantation, also needs to be examined.

In conclusion, our findings suggest that periodontal pathogen-induced intestinal dysbiosis involving an increase in the abundance of SCFA-producing genera may affect transplant immunity by increasing SCFA levels and the proportion of Tregs.

## Materials and methods

### Ethical statement

All experimental protocols were approved by the Animal Care and Use Committee of Kyushu University (approval number: A21-061). Mouse handling and experimental procedures were performed in compliance with the Principles of Laboratory Animal Care^47^.

### Animals

This study used 5- to 6-week-old C57BL/6 J male mice (SLC Japan Inc., Tokyo, Japan) as skin graft recipients. The donor mice were 5- to 6-week-old B6D2F1 male (SLC Japan Inc., Tokyo, Japan). All mice were housed under specific pathogen-free conditions and had free access to food and water.

### Preparation and application of *Pg*

*Pg* strain W83 was grown at 37 °C under anaerobic conditions for 7 days on brain heart infusion agar plates (Becton Dickinson, New Jersey) supplemented with 10% defibrinated horse blood, 5 mg/mL hemin, and 0.5 µg/mL menadione (AnaeroPack system; Mitsubishi Gas Chemical, Tokyo). Colonies were then selected and anaerobically subcultured at 37 °C for 48 h in brain heart infusion broth supplemented with hemin and menadione. *Pg* turbidity (OD 0.8) was spectrophotometrically determined at 600 nm, and an equal amount of the bacterial slurry was mixed with 4% sodium carboxymethylcellulose (CMC) for administration to the mice (Wako Pure Chemical Industries, Tokyo; Japan).

### Oral administration of *Pg*

The murine experimental periodontitis model was established according to Arimatsu et al^18^. All mice were given 1 mg/mL sulfamethoxazole and 200 µg/mL trimethoprim in drinking water for 7 days to reduce their native oral flora^48^. Mice were randomly assigned into two groups by a computer-generated random number table: the Pg and control groups. Mice in the Pg group were given a total of 10^9^ CFUs of live *P. gingivalis* in 100 µL PBS with 2% carboxymethyl cellulose via oral gavage twice per week. The number of bacteria administered was determined by considering mouse body weight and the number of bacteria in the saliva of patients with periodontitis. Mice in the control group received PBS (an equal amount of PBS mixed with 4% CMC) orally twice per week. Mice in both groups continued receiving oral administration of *Pg* or PBS until they were sacrificed. All evaluations were performed by one examiner (M. T.), who was blinded in terms of which treatment was given, *Pg* or PBS administration.

### Analysis of the microbiota

Mouse cecal feces were frozen at − 80 °C and sent to Techno Suruga Laboratory (Shizuoka, Japan) for analysis. DNA extraction was performed as previously described^49^ using an automated DNA isolation system (GENE PREP STAR PI-480 KURABO, Japan). The V3–V4 regions of bacterial and archaeal 16S rRNA were amplified using Pro341F/Pro805R primers and the dual-index method^49,50^. Barcoded amplicons were paired-end sequenced using a 2 × 301-bp cycle on an MiSeq system with MiSeq Reagent Kit version 3 (600 Cycle) chemistry. The primer sequences were trimmed from the paired-end sequencing reads using Cutadapt ver. 1.18 with default settings^51^. Paired-end sequencing reads were then merged using the fastq-join program with default settings^52^. Only joined reads that had a quality value score of ≥ 20 for more than 99% of the sequence were extracted using FASTX-Toolkit^53^.

For the diversity analysis, the joined amplicon sequence reads were processed using QIIME2 ver. 2020.2^54^. Quality filtering and deletion of chimeric sequences were performed, and then representative sequences were created using DADA2 (Divisive Amplicon Denoising Algorithm 2) denoise-single plugin ver. 2017.6.0 with default settings^55^. The taxonomy of representative sequences was assigned with the Silva database by training a Naïve Bayes classifier using the q2-feature-classifier plugin. Alpha diversity indices (observed features) were calculated alpha-rarefaction plugin. β diversity was analyzed by unweighted UniFrac using the core-metrics-phylogenetic plugin. The Emperor tool was used to generate the principal coordinates analysis plots. The statistical significance of the similarity of bacterial communities between the groups was assessed with the analysis of similarities test using the beta-group-significance plugin.

### Measurement of SCFAs

Mouse cecal feces were frozen at − 80 °C and analyzed by Techno Suruga Laboratory (Japan). The amount of SCFAs in the feces was quantified following a modified method previously described by García-Villalba^56^. To evaluate SCFAs, 0.1 g of feces was placed in a 2.0 mL tube with zirconia beads and suspended in 0.9 mL 0.5% phosphoric acid. Each sample was heated at 85 °C for 15 min and vortexed at 5 m/s for 45 s using FastPrep 24 (MP Biomedicals, CA, USA). Then, 0.4 mL of the supernatant was transferred to 1.5 mL tube, mixed with 0.4 mL ethyl acetate, and shaken for 30 min. Finally, 0.2 mL of the supernatant was mixed with 1 mM 4-methyl valeric acid as an internal standard.

A flame ionization detector (7890B, Agilent Technologies, USA) and a capillary column DB-WAXetr (30 m, 0.25 mm id, 0.25 µm film thickness, Agilent Technologies, USA) were used to measure SCFAs in feces by gas chromatography. Helium was used as the carrier gas at a rate of 1.2 mL/min. The detector temperature was kept at 250 °C. The oven temperature program was as follows: 50 °C; then 10 °C/min to 90 °C; 15 °C/min to 150 °C; 5 °C/min to 170 °C; 20 °C/min to a final temperature of 250 °C, held for 4 min. Acetate, propionate, butyrate, valerate, and caproate were measured.

### Flow cytometry

Hemolyzed peripheral blood samples or spleen cells were incubated with purified CD16/32 (cat#553142 BD Pharmingen (BD), San Jose, CA) to block nonspecific staining. Dead cells were defined as cells that were positive for Fixable Viability Stain 780 (cat# 565388 BD). Tregs that differentiated from naïve CD4+ cells were identified by a two-step staining process. First, cells were surface-stained with APC-anti-CD25 antibody (cat# 561048 BD) and FITC-anti-CD4 antibody (cat# 557667 BD). Subsequently, the cells were fixed and permeabilized with Mouse Foxp3 Buffer Set (cat#560409 BD), and then stained with PE anti-mouse Foxp3 antibody (cat# 560414 BD). Cell samples were analyzed on a BD FACS VERSE flow-cytometer (BD). Gating strategy was shown in Supplemental Fig. S2.

### Skin transplantation

After 6 weeks of oral administration of *Pg* or PBS, 1 cm^2^ tail skin patches harvested from donor mice were transplanted onto the backs of the recipient mice. All procedures were performed on mice anesthetized by intraperitoneal injection of a mixture of medetomidine, midazolam, and butorphanol. Skin grafts were visually inspected and considered to be rejected when less than 50% of the graft remained viable.

### Histology

The skin specimens were fixed with 4% paraformaldehyde, dehydrated, and embedded in paraffin. The specimens were cut into 4 µm sections. Slides containing fixed tissue were stored in 70% ethanol prior to processing and staining with H&E. A microscope (Keyence 800) was used to assess inflammation of the skin graft. The percentage of inflammatory cells in the sections was automatically calculated using a hybrid cell count application (BZ-H4C, KEYENCE, Osaka, Japan) with BZ-X Analyzer software (BZ-H4A, KEYENCE).

### Statistical analysis

Data are presented as the mean ± standard deviation for normally distributed continuous variables, as the median (range) for continuous variables that were not normally distributed, and as a number for categorical variables. Student’s t-test was used to analyze normally distributed continuous variables, and the Mann–Whitney U test was used to analyze continuous variables that were not normally distributed. Kaplan–Meier analysis was used to calculate skin graft survival, and the log-rank test was used to evaluate differences between curves. A *p*-value of < 0.05 was considered statistically significant, and all statistical analyses were performed using R software version 4.22 (R Project for Statistical Computing, https://cran.ism.ac.jp/) and Graphpad Prism 7.03.

#### Statement on ARRIVE guidelines

Study was conducted in accordance with ARRIVE guidelines.

## Supplementary Information

Supplementary Figure S1.

Supplementary Figure S2.

## Data Availability

The datasets used and/or analyzed during the current study available from the corresponding author on reasonable request.
