# Supplementary figures and images for: Effects of periodontal pathogen-induced intestinal dysbiosis on transplant immunity in an allogenic skin graft model
Source: Sci Rep. 2023 Jan 11;13:544. doi: 10.1038/s41598-023-27861-4 (PMC9834409; doi:10.1038/s41598-023-27861-4)

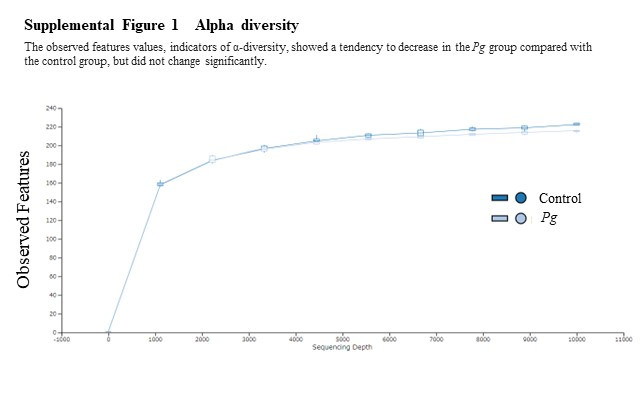

Supplement: Supplementary file 1 — Supplementary Figure S1. [file 41598_2023_27861_MOESM1_ESM.jpg]

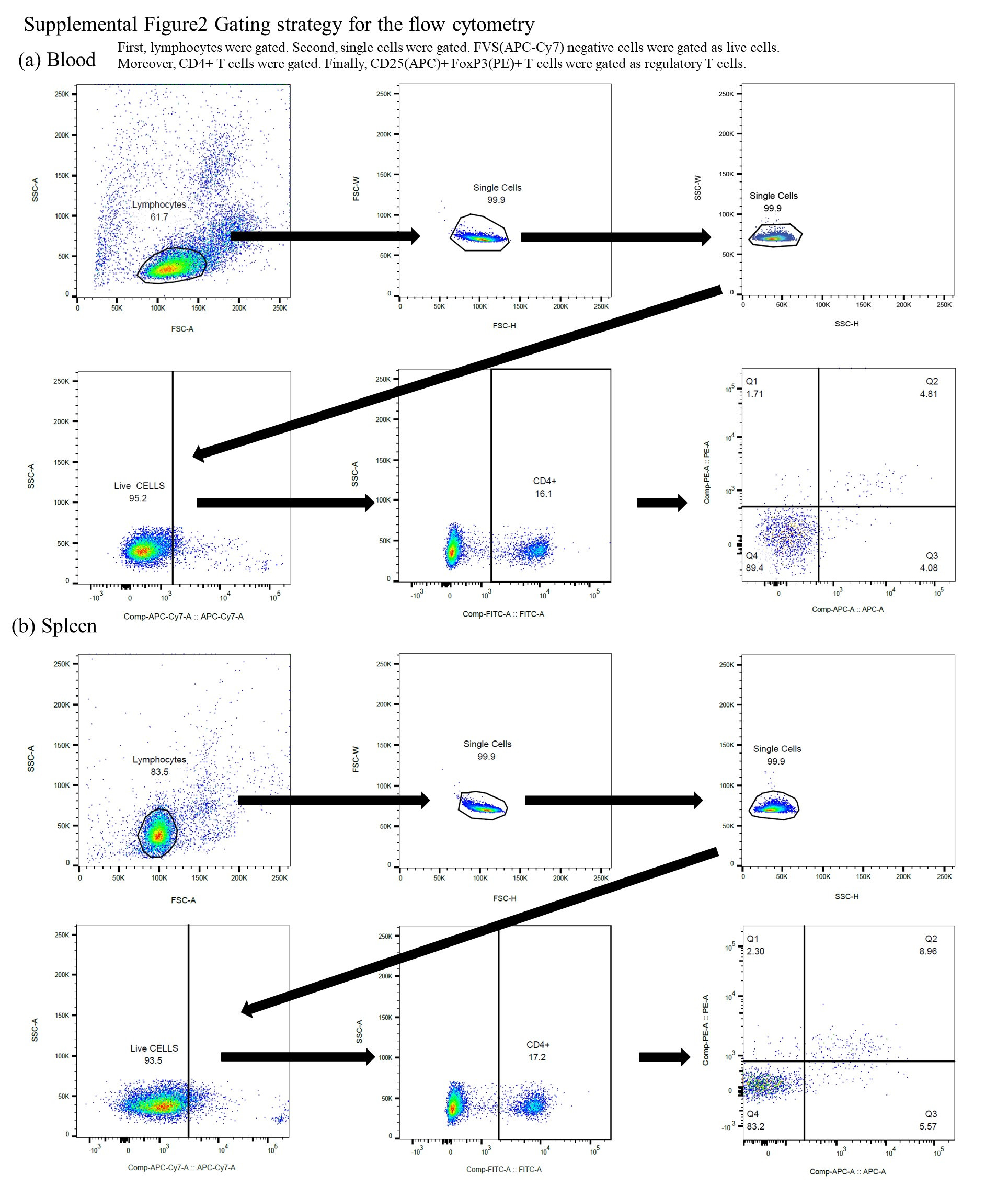

Supplement: Supplementary file 2 — Supplementary Figure S2. [file 41598_2023_27861_MOESM2_ESM.jpg]
